# Rapid and Precise Detection of Nickel(II) Ions Using a Fluorescent Polymeric Sensor

**DOI:** 10.1007/s10895-025-04439-z

**Published:** 2025-07-01

**Authors:** Soner Çubuk, Tutku Demirtaş, Belma Gjergjizi Nallbani, Memet Vezir Kahraman

**Affiliations:** 1 Department of Chemistry, Faculty of Sciences, Marmara University, 34722 Istanbul, Türkiye; 2 Faculty of Pharmacy, UBT – Higher Education Institution, Lagjia Kalabria, 10000 Pristina, Republic of Kosovo

**Keywords:** Selective Ni(II) detection, Wastewater, Blood serum, Polymer-based membrane, Fluorometric sensor

## Abstract

In this research, a polymeric fluorescence sensor was developed to detect Ni(II) ions. A polymeric membrane was prepared, and its structural and morphological properties were analyzed through FTIR and SEM. The fluorescence properties of the sensor and the optimal conditions for Ni(II) ion detection were investigated. The sensor's excitation and emission wavelengths were identified as 390 nm and 530 nm, respectively, with a reduction in fluorescence intensity observed in the presence of Ni(II) ions. Key factors affecting fluorescence intensity, such as pH, response time, and potential interference from foreign ions, were extensively studied. Optimal conditions were determined to be at pH 5.0 and 20 s. The method demonstrated a linear detection range of 1.70 × 10⁻⁸—3.41 × 10⁻⁷ mol L^−1^ and a detection limit of 4.81 × 10⁻⁹ mol L^−1^. This method has been successfully applied for the selective, sensitive, and rapid quantification of Ni(II) ions in wastewater, blood serum, and tap water. It is a method with the potential to offer significant advantages over existing literature methods, including high sensitivity, selectivity, low cost, and an environmentally friendly analytical approach.

**Supplementary Information:**

The online version contains supplementary material available at 10.1007/s10895-025-04439-z.

## Introduction

Nickel is a widely utilized transition metal in industrial, agricultural, and technological applications [1], including alloy production, electroplating, and catalysis [2, 3]. Even though nickel is essential, high concentrations of it can be harmful to human health and the environment [4]. Long-term exposure to nickel can raise the risk of lung cancer and cause allergic reactions and respiratory problems, especially in industrial settings. Due to frequent exposure to nickel dust and fumes, workers handling nickel are particularly vulnerable [5]. Furthermore, by damaging plant, microbial, and aquatic life in both terrestrial and aquatic environments, nickel mining and use contaminate soil, water, and air, causing ecosystem disruption and biodiversity loss. Therefore, it is essential to detect Ni(II) ions in biological and environmental samples in order to protect ecosystems and public health [6]. However, traditional analytical methods, such as atomic absorption spectrometry (AAS) [7] and inductively coupled plasma mass spectrometry (ICP-MS) [8], are accurate and sensitive; however, they often require expensive equipment and extensive sample preparation. These limitations make it clear that we need alternative methods to find things that are easy, quick, and inexpensive.

Fluorometric sensors have gotten much attention in the last few years as a possible new way to find metal ions because they are very sensitive, selective, and respond quickly [9, 10]. These sensors can detect very small amounts of a substance with minimal sample volume and without needing complicated equipment by using fluorescence as the transduction mechanism [11]. Polymeric membranes, in particular, have become a good base for developing sensors because they can be changed chemically, are structurally stable, and can do many different things. Adding fluorescence-active parts to polymeric membranes makes it possible to make sensors that can interact with specific ions [12].

Polymer-based fluorometric sensors offer a novel approach for detecting extremely small amounts of Ni(II) ions with high sensitivity and selectivity [13, 14]. Sensors use the interaction between nickel ions and functional fluorophores in a polymeric matrix to cause fluorescence changes that can be measured [15]. This method offers many benefits that make it highly effective in various applications and streamlines the detection process by reducing the need for sophisticated instrumentation [16]. They can be used on a large scale because they are also economical, utilizing reasonably priced materials and fabrication techniques. A range of analytical studies, including spectral studies, has been conducted to optimize its performance in terms of excitation and emission wavelengths, detection limits, and selectivity towards interfering ions. The as-fabricated sensor demonstrates high prospects for practical application in Ni detection, offering good performance and serving as a cost-effective and convenient method compared to traditional assays. Many of them are made of eco-friendly materials, which aligns with sustainable practices, and they are portable and lightweight, making on-site analyses easier [17, 18]. These combined characteristics establish polymer-based fluorometric sensors as indispensable tools in industrial monitoring, biomedicine, and environmental science. The goal of this work is to develop and evaluate a fluorometric polymer-based sensor specifically suited for Ni(II) ion selective detection. Using a polymeric membrane as a matrix, the sensor incorporates functional moieties that, when in contact with Ni(II), either quench or enhance fluorescence. The sensor's analytical features, such as excitation and emission wavelengths, detection limits, and selectivity over interfering ions, have been systematically optimized through investigations of spectral properties. The prepared sensor demonstrates considerable promise in addressing the challenges associated with nickel detection, offering a low-cost and portable alternative to conventional techniques.

This research advances environmental monitoring and safeguards public health by providing an efficient and reliable approach to nickel detection. It also paves the way for further innovations in polymer-based sensor technology.

## Materials and Methods

### Instruments and Reagents

The study utilized advanced equipment for precise measurements and analysis. A Varian Carry Eclipse spectrofluorometer was used for fluorescence studies, and a Precisa XB 220 A SCS balance ensured accurate weighing. Ultrapure water was obtained from the Millipore Direct-Q®−3 system, while a Velp ARE magnetic stirrer aided solution preparation. A WTW inoLab® pH 7110 m measured pH, and a 300 W OSRAM UV lamp facilitated polymer curing. Materials that needed to be dissolved were processed and homogenizing using a Bandelin Sonorex ultrasonic bath, and membranes were dried with an Alpha 1–2 LO lyophilizer. Structural analysis was performed using a Perkin-Elmer ATR-FTIR spectrophotometer, and morphological studies employed a Philips XL30 SEM.

Furthermore, analytically pure reagents were utilized, and working solutions were prepared by diluting stock solutions to the required ratios. Key chemicals, including Polyethylene glycol diacrylate (PEGDA), trimethylolpropane triacrylate (TMPTA), 2,5-dimercapto-1,3,4-thiadiazole (2-SH), pentaerythritol tetrakis(3-mercapto propionate) (4-SH), camphorquinone (CQ), and Darocure 1173, were sourced from Sigma Aldrich (Bo-Ga, Turkey). pH regulation was achieved using buffer solutions prepared with potassium chloride (KCl), hydrogen chloride (HCl), sodium acetate trihydrate (NaCH_3_COO·3H_2_O), acetic acid (CH_3_COOH), sodium dihydrogen phosphate monohydrate (NaH_2_PO_4_·H_2_O), and dipotassium hydrogen phosphate (K_2_HPO_4_), following the Britton-Robinson buffer system [19].

### Solution Preparation and Characterization

#### Ni(II) Standard Solutions Preparation

A stock Ni(II) solution with a concentration of 1000 ppm (S-1) was diluted to prepare an intermediate solution (S-2) with a concentration of 1 ppm Ni(II). Standard solutions for the experiments were obtained using the appropriate volumes of the S-2 solution. Buffer solutions specific to each solution set were used to dilute the prepared solutions to their final volumes. Consequently, 48 standard solutions covering a Ni(II) concentration range of 1.7 × 10⁻⁸ M to 3.4 × 10⁻⁷ mol L^−1^ were prepared across eight different pH buffer solutions, with each set containing six standards. To protect these solutions from light, they were wrapped in aluminum foil and stored at +  4 °C in a refrigerator. Before the experiments, all solutions were allowed to reach room temperature.

#### Preparation of Solutions for Investigating the Effects of Foreign Species

To investigate the effects of foreign species, solutions were prepared to examine the influence of potential ions (Fe^3^⁺, Hg^2^⁺, Cu^2^⁺, Co^2^⁺, Cr^3^⁺, Al^3^⁺, Cd^2^⁺, Mn^2^⁺, Zn^2^⁺, Pb^2^⁺, Au^3^⁺, Ag⁺, Ba^2^⁺, Mg^2^⁺) on the fluorescence intensity of the polymeric membrane in the presence of Ni(II). The concentration of each ion was up to 1000 times higher than that of Ni(II). Measurements of fluorescence intensity determined tolerable foreign ion levels with a signal deviation of ± 5% were considered acceptable. Protective measures for Ni(II) solutions, including shielding from light, were consistently applied to ensure stability.

#### Preparation of Polymer-based Fluorescence Membrane Sensor

**Scheme 1:**
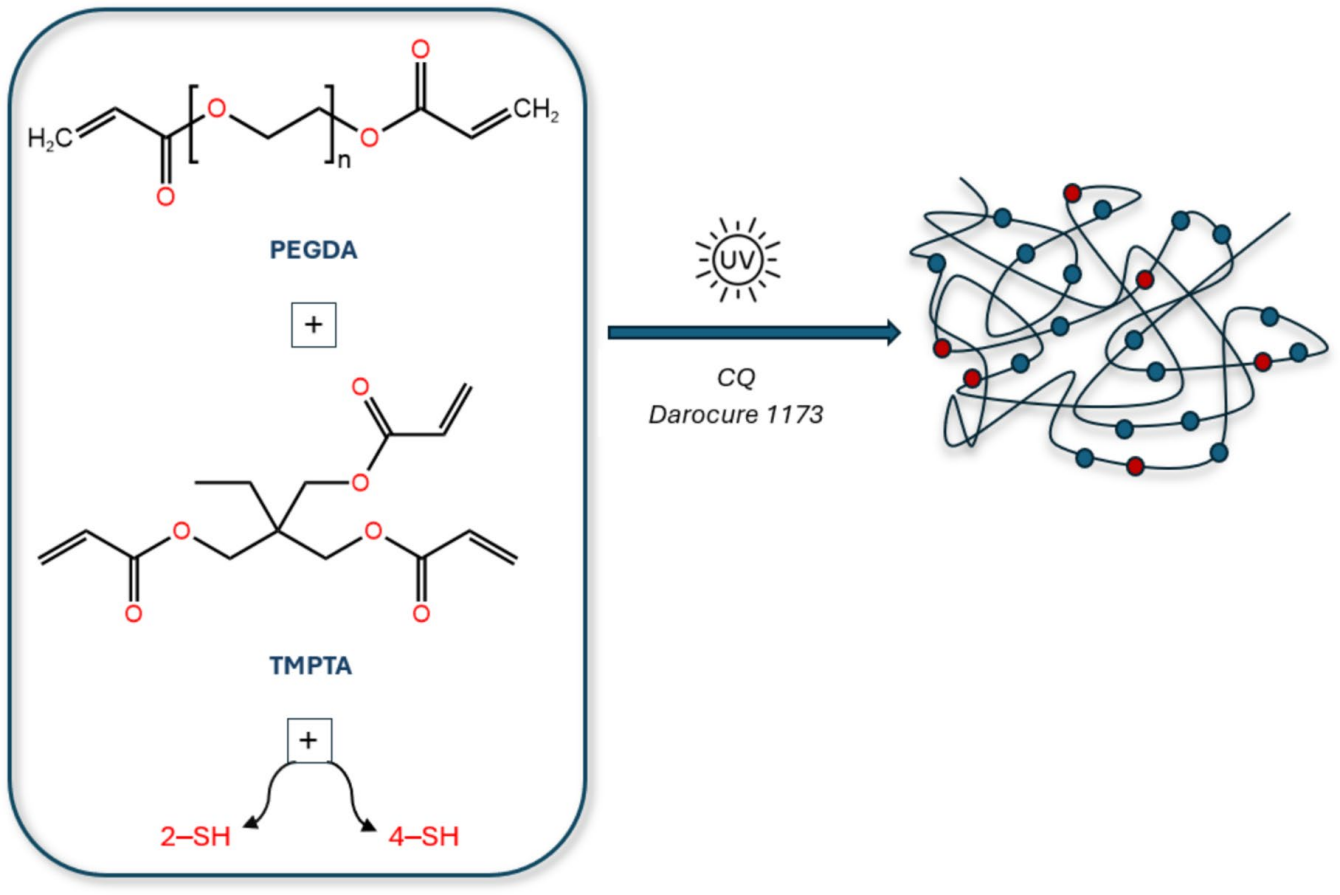
Structure of the cross-linked polymer

The optimal formulation for the polymeric membrane was identified, avoiding issues such as incomplete drying, wrinkling, excessive swelling in aqueous media, inadequate interaction with solutions, or disintegration at varying pH levels. This formulation was used in subsequent stages with specific monomer ratios. %60 PEGDA, 40% TMPTA, 1% 2-SH, and 20% 4-SH were precisely weighed, shielded from light, and homogenized. 1% Camphorquinone (CQ) and 1% 2-hydroxy-2-methylpropiophenone (Darocure 1173) were photoinitiators. The mixture was cast into a Teflon® mold using a Pasteur pipette and cured under UV light. Scheme 1 shows the structure of the cross-linked polymer. The membranes were immersed in ultrapure water overnight to remove residues and dried in a lyophilizer for further use. The surface area of ​​the obtained membranes is 4.8 cm^2^ and their thickness is 1.0 mm. The SI (Supportive Information) section provides a detailed preparation procedure.

#### SEM and ATR-FTIR Characterization

The morphological properties of the polymeric-based membranes prepared within the scope of this study were determined using the Philips XL30 model ESEM-FEG device. Furthermore, the functional groups were characterized with an ATR-FTIR spectrophotometer manufactured by Perkin-Elmer.

## Results and Discussion

### Spectral Analysis of the Membrane Using ATR-FTIR

**Fig. 1:**
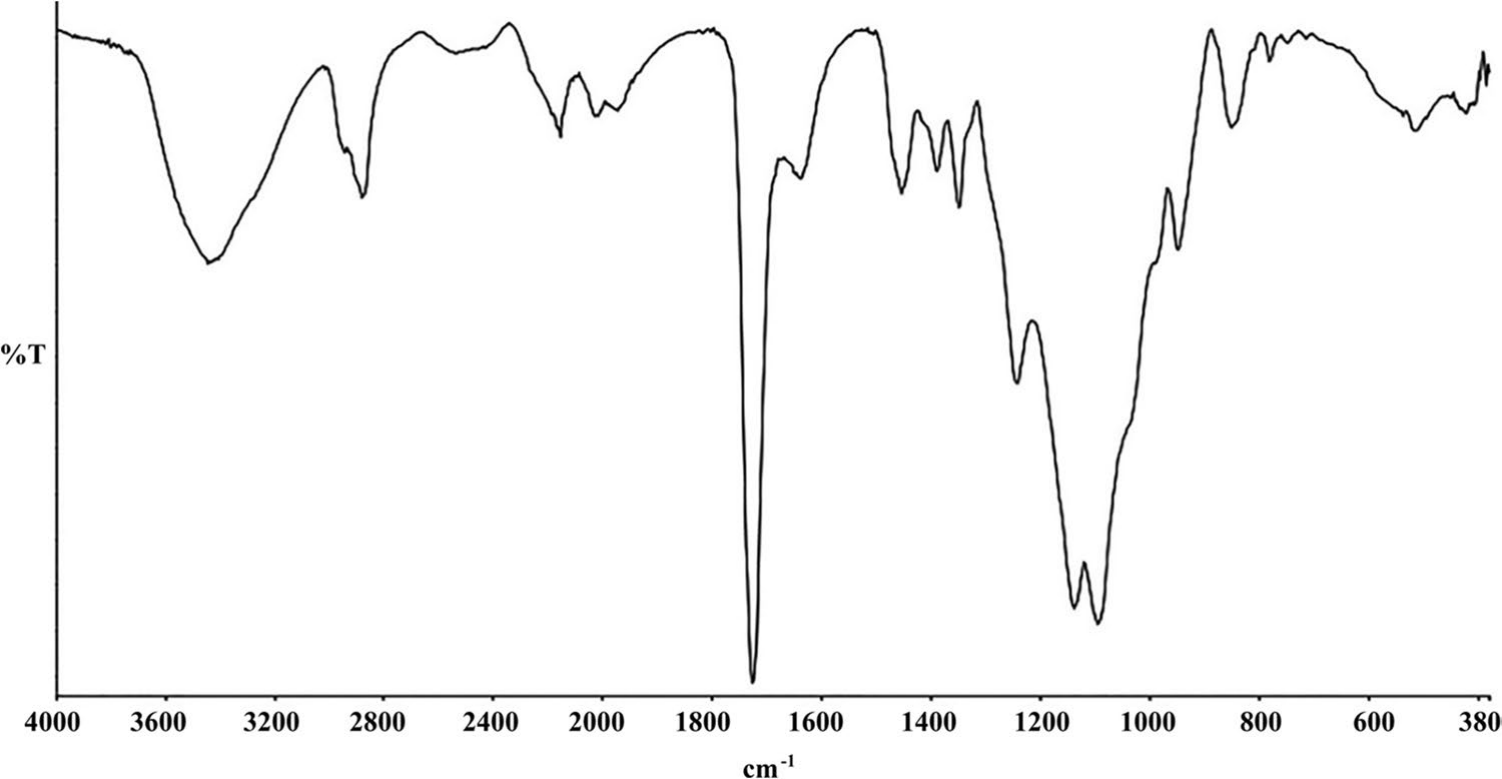
ATR-FTIR spectrum of the polymer-based membrane

The ATR-FTIR spectrum of the polymeric membrane, obtained through scans in the wavelength range of 380–4000 cm⁻^1^, is presented in Fig. 1. The peaks observed at 2949 cm⁻^1^ and 2883 cm⁻^1^ correspond to the asymmetric -C-H stretching vibrations associated with TMPTA and PEGDA. The absorption band at 1727 cm⁻^1^ is attributable to the carbonyl groups in PEGDA, TMPTA, and 4-SH. The peak near 1100 cm⁻^1^ signifies C-O stretching vibrations in PEGDA. The band at 1389 cm⁻^1^ and 1637 cm⁻^1^ indicates vibrations C = N of the thiadiazole ring. The disappearance of the characteristic peak at 1619 cm⁻^1^, corresponding to the acrylate group, as well as the loss of the S–H stretching peak at 2476 cm⁻^1^, in conjunction with the emergence of a new S–S stretching peak at 513 cm⁻^1^, provides strong evidence for the occurrence of crosslinking within the polymeric structure [20–22].

### Surface morphology (SEM imaging) characterization

**Fig. 2:**
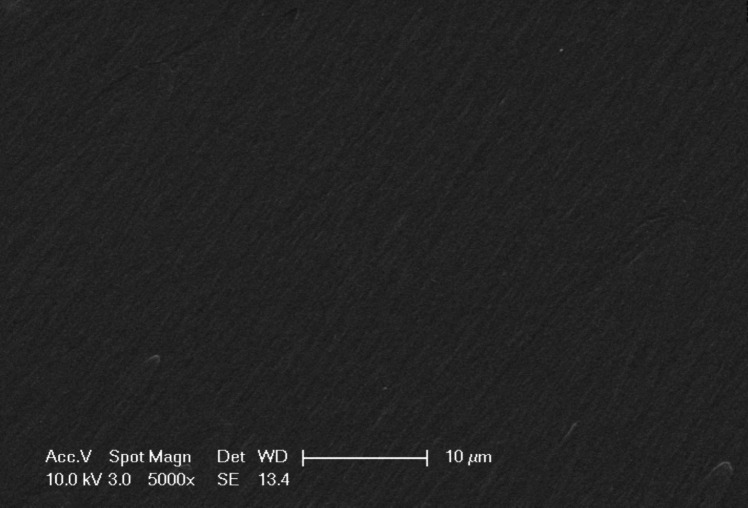
SEM image of the polymeric membrane (5000 ×)

The surface morphology of the prepared membrane sensor was analyzed using Scanning Electron Microscopy (SEM). As illustrated in Fig. 2, the SEM image captured at 5000 × magnification revealed that the crosslinked membrane exhibited a smooth, non-porous structure. In addition, the gel content (6 h, methyl ethyl ketone extraction) was 99.7%, confirming that a highly cross-linked polymeric membrane was constructed. Furthermore, the membrane was observed to possess a homogeneous morphology, free from cracks and fractures, which confirms its structural integrity and uniformity.

### Investigation of the Suitability of Polymer-Based Sensors for Ni(II) Analysis

#### Sensor Spectral Characterization Study

**Fig. 3:**
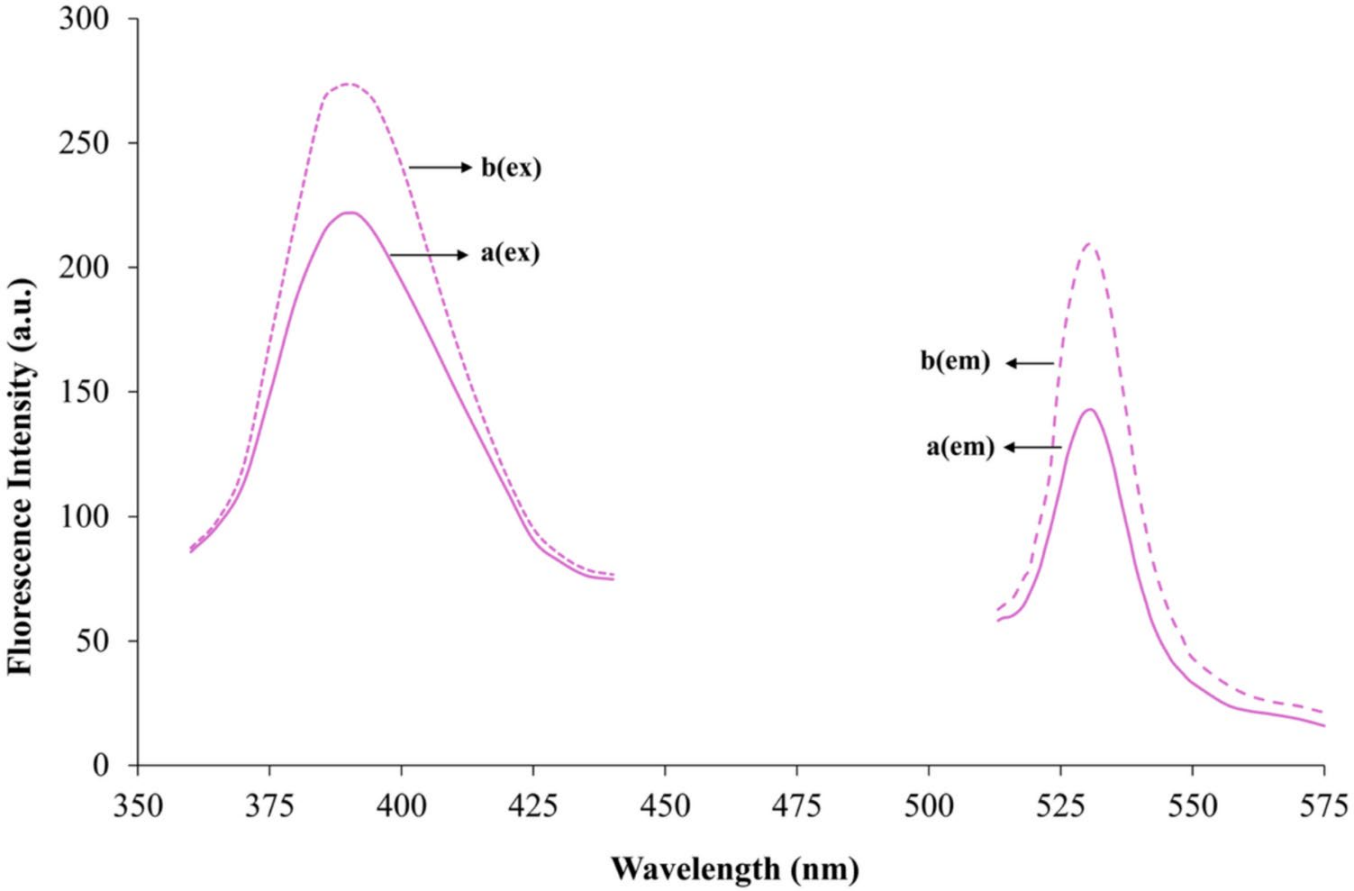
**a** Excitation and emission intensity spectra of the sensor in the presence of 1.19 × 10⁻⁷ mol L^−1^ Ni(II) (line), and (**b**) in its absence (dot line) are shown

A spectral scan was conducted to determine the excitation and emission wavelengths of the polymeric membrane in the presence of 1.19 × 10⁻⁷ mol L^−1^ nickel. During the scan, the optimal excitation and emission slit widths, as well as the photomultiplier tube voltage, were identified. The excitation and emission wavelengths were determined to be 390 nm and 530 nm, respectively, with optimal slit widths of 10 nm and a photomultiplier tube voltage of 600 V. The corresponding spectrum is presented in Fig. 3. The thiadiazole ring, thioether groups and carbonyl functional components (lone-pair electrons) may responsible for fluorescence. The unusual strong fluorescence is explained by various possible factors, i.e., the extended π system due to the polymeric structure, the somewhat stiff ester linkage due to the steric hindrance and hydrogen bonding.

The results indicated a decrease in fluorescence intensity in the presence of Ni(II) ions. The decrease in fluorescence intensity is thought to be due to the ability of Ni(II) ions to form coordinated complexes with high affinity thiol (-SH) groups and thiadiazole rings in the membrane structure, and the formation of this complex disrupts the electronic structure of fluorescent chromophores or causes nonradiative deactivation of their excited states. Additionally, ester and ether groups may contribute to this situation through weaker interactions. The sensor exhibits excellent photostability, showing no degradation or performance loss upon long-term UV exposure.

#### Identifying the Optimal pH Level for the Developed Sensor

To investigate the effect of pH on fluorescence intensity in the presence of Ni(II) ions for the synthesized polymeric sensor, buffer solutions with pH values ranging from 1.0 to 8.0 were prepared. These buffers were mixed with 1.19 × 10⁻⁷ mol L^−1^ Ni(II) ion solutions, and their fluorescence intensities were measured. The results were plotted as fluorescence intensity against pH (Figure S1, in the SI section). The graph analysis revealed that the fluorescence intensity reached its maximum at pH 5.0, increasing within the pH range of 1.0–5.0 and decreasing beyond pH 5.0. The decrease in fluorescence intensity at pH values ​​lower than pH 5.0 is thought to be due to the penetration of H^+^ ions into the membrane and thus the protonation of nitrogen atoms in the thiadiazole ring and the decrease in the mobility of π-electrons in the conjugated double bond system.

On the other hand, the decrease in fluorescence intensity at pH values ​​higher than pH 5.0 is interpreted as the formation of hydroxide of Ni(II) ions as well as a slight swelling of the polymeric membrane. The results were found to be consistent with the literature [23].

As a result of the study, with six repetitions, a pH value of 5.00 ± 0.02 was determined to be the optimal pH for this sensor.

#### Determining the Response Time for the Developed Sensor

The temporal variation in fluorescence intensity of the sensor was examined in the presence of 1.19 × 10⁻⁷ mol L ^1^ Ni(II) ions. During this study, the pH value was kept constant at pH: 5.0 with the acetate buffer system. Measurements were conducted at intervals of 5 s over a total duration of 200 s. The graph in Figure S2 (in the SI section) indicates that fluorescence intensity increased during the initial 20 s, followed by a distinct decline from 20 to 200 s. The increase in fluorescence intensity of the membrane sensor observed during nickel ion determination, subsequent stabilization, and final signal intensity reduction can be attributed to two main mechanisms. The signal reduction is thought to be due to possible back-diffusion of nickel ions from the membrane to the solution, as well as additional interactions such as the internal filter effect and/or physical blockages on the sensor surface that may occur after the sensor's quenching capacity reaches saturation. The peak fluorescence intensity, observed at the 20th second, was selected as the optimal point for analysis. Accordingly, a waiting period of 20 s was determined to be sufficient for analytical purposes within the scope of this method.

#### Determination of the Calibration Range, Limit of Detection

**Fig. 4:**
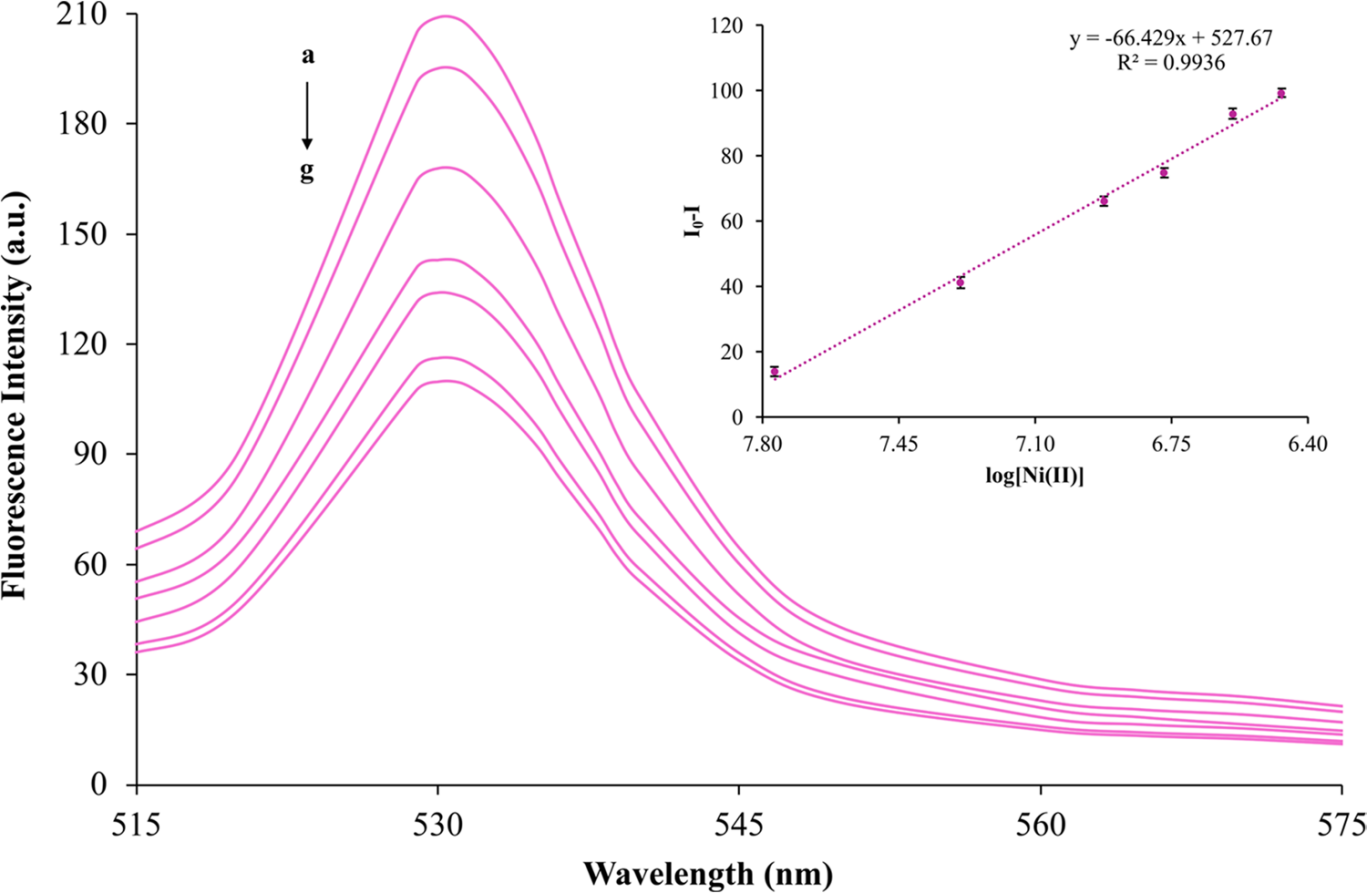
Main Graph: Fluorescence spectra of Ni(II) standard solutions obtained using an excitation wavelength of 390 nm. Inset Graph: Calibration curve prepared using standard solutions (λ_ex_/λ_em_: 390 nm/530 nm; pH: 5.0; t: 20 s)

Under the optimized experimental conditions (excitation wavelength: 390 nm; emission wavelength: 530 nm; duration: 20 s), fluorescence intensity measurements were performed for six standard solutions of Ni(II) within the concentration range of 1.70 × 10⁻⁸ to 3.4 × 10⁻⁷ mol L^−1^ in a pH 5.0 buffer system. Emission spectra were obtained concurrently with calibration curves, which are presented in Fig. 4. In this figure, the fluorescence intensity in the presence of Ni(II) ions (“I”) and their absence (“I₀”) is depicted. The standard solutions were prepared with the following Ni(II) concentrations: (a) 0 mol L^−1^, (b) 1.70 × 10⁻⁸ mol L^−1^, (c) 5.11 × 10⁻⁸ mol L^−1^, (d) 1.19 × 10⁻⁷ mol L^−1^, (e) 1.70 × 10⁻⁷ mol L^−1^, (f) 2.56 × 10⁻⁷ mol L^−1^, and (g) 3.41 × 10⁻⁷ mol L^−1^. The method's detection limit (LOD) was determined using a reference pH 5.0 acetic acid/sodium acetate buffer system. The fluorescence intensity of 7 solutions containing 1.70 × 10^–8^ mol L^−1^ Ni(II) was measured under optimum operating conditions for the limit of detection (LOD) and limit of quantification (LOQ) of the sensor.

LOD and LOQ values were calculated using the calibration graph's standard deviation value (SD) and slope (m). LOD and LOQ values were calculated using the following formulas:

$$LOD= 3\sigma /m$$

$$LOQ= 10\sigma //m$$

The LOD and LOQ for this method were determined to be 4.81 × 10⁻⁹ mol L⁻^1^ and 1.60 × 10⁻⁹ mol L⁻^1^, respectively.

#### Sensor Regeneration and Reusability

**Fig. 5:**
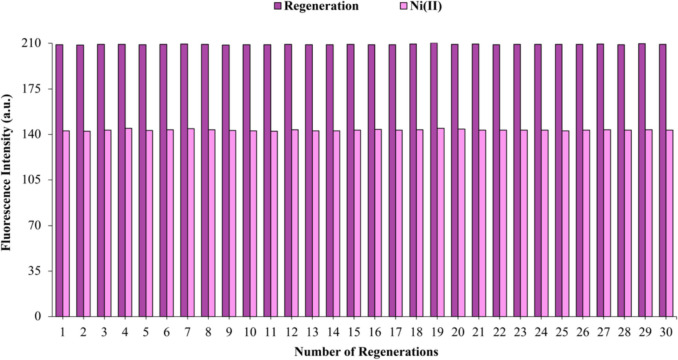
Results of fluorescence intensity changes following the sensor's exposure to Ni(II) ions and its regeneration using deionized water and a pH: 5.0 buffer (Purple Column: Regeneration; Pink Column: 1.19 × 10⁻⁷ mol L^−1^ Ni(II))

To restore the initial fluorescence intensity of the sensor, a regeneration procedure was performed that involved a 1-min rinse with deionized water, followed by a 30-s wash with a pH 5.0 buffer solution. This process proved effective in reinstating the original fluorescence intensity. Furthermore, after exposure to Ni(II) ions, the sensor was found to maintain its functionality for at least 150 reuse cycles using this regeneration method. Figure 5 illustrates the results from 30 regeneration cycles. The standard deviation of the fluorescence intensity values between the initial and 150th readings was calculated as (± 1.62). The average fluorescence intensity of the membrane suffered a signal loss of 0.2% during the measurement period. This extremely low percentage, combined with the reported standard deviation, further confirms the long-term stability of our membrane. Since it was determined that there was no significant change after the 30th measurement, the graph is provided up to the 30th measurement.

#### Sensor Durability, Stability, and Reproducibility

The polymeric sensor membrane was stored in the dark within a desiccator, and fluorescence intensity measurements over six months showed no deviations exceeding ± 5% from the initial value. For short-term stability, the fluorescence intensity in the presence of 1.19 × 10⁻⁷ mol L^−1^ Ni(II) was measured under set conditions for 12 h at 10-min intervals, with a standard deviation of ± 1.25%. Repeatability was assessed using five membranes with identical formulations under defined conditions, yielding a standard deviation of ± 3.35%.

Six repetitions were made in 3 different concentrations (4.26 × 10^–8^, 8.52 × 10^–7^, 1.70 × 10^–7^ mol L^−1^) for recovery/accuracy tests. The results showed that Ni(II) ions had acceptable recoveries with mean values of 101.55% and 102.43% for inter-day and intra-day repeatability, respectively. A precision study of the method was conducted as intra-day and inter-day repeatability. Intra-day repeatability for Ni(II) ions, including three concentration levels, relative standard deviations (RSD%) were between 0.42% and 0.71%, while inter-day relative standard deviations (RSD%) were 0.38% and 0.55%.

### Investigation of the Impact of Foreign Species

Defining the parameters for analysis is essential, as certain elements can interfere with the environment surrounding the target metal [24]. Therefore, various ions'fluorescence intensity measurements were performed to evaluate interference during Ni(II) determination. For each interfering ion, we performed stepwise measurements in the presence of 1.19 × 10⁻⁷ mol L^−1^ Ni(II) ions, increasing the amounts of interfering ions from 10 mol-fold to 1500 mol-fold relative to Ni(II). The values reported in Table 1 represent the highest concentration of each interfering ion at which no significant interference was detected during these stepwise tests. Considering that the acceptable upper limit value may be the concentration value for which there is a maximum 5% change in fluorescence intensity, the acceptable upper limits are determined for ions. These detailed data demonstrate the robust selectivity and stability of our sensor in detecting Ni(II) over a wide range of interfering ion concentrations. The sensor demonstrated high selectivity, allowing interference-free analysis even with 900-fold excesses of Co^2+^, Cu^2+^, and Hg^2+^, and 1100-fold excess of Fe^3+^, highlighting its robustness for Ni(II) determination.

**Table 1:** The permissible concentration levels of potential interfering ions (1.19 × 10⁻⁷ mol L^−1^ Ni(II))

Foreign ion	Permissible upper limit (mol L^−1^)
Fe^3+^	1.31 × 10^–4^
Hg^2+^	1.07 × 10^–4^
Cu^2+^	1.07 × 10^–4^
Co^2+^	1.07 × 10^–4^
Cr^3+^	1.31 × 10^–4^
Al^3+^	1.31 × 10^–4^
Cd^2+^	1.19 × 10^–4^
Mn^2+^	1.19 × 10^–4^
Zn^2+^	1.07 × 10^–4^
Pb^2+^	1.07 × 10^–4^
Au^3+^	1.07 × 10^–4^
Ag^+^	1.31 × 10^–4^
Ba^2+^	1.90 × 10^–4^
Ca^2+^	1.79 × 10^–4^
Mg^2+^	1.79 × 10^–4^

### Analytical Uses of the Developed Sensor

For this purpose, certified reference solutions, including wastewater (SPS-WW-1, SPS-WW-2) and blood serum (Seronorm Trace Elements Serum Level-1, Seronorm Trace Elements Serum Level-2), were diluted appropriately with a pH 5.0 buffer solution. The analysis results were found to be consistent with the certified values. Additionally, recovery studies were conducted using tap water samples spiked with three different concentrations of Ni(II). The findings suggest that the developed method offers a viable alternative to Ni(II) determination techniques Table 2.

**Table 2:** The results and relative errors (95% confidence levels, *n* = 6) for certified reference materials were analyzed using the developed method

Sample	Certificate Value (mol L^−1^)	Current Study (mol L^−1^)	Relative Error (%)
SPS-WW1	(1.70 ± 0.01) × 10^–5^	(1.66 ± 0.12) × 10^–5^	2.41
SPS-WW2	(8.52 ± 0.04) × 10^–5^	(8.33 ± 0.09) × 10^–5^	2.29
Seronorm Trace Elements Serum Level-1	(9.88 ± 1.19) × 10^–8^	(9.51 ± 0.27) × 10^–8^	3.89
Seronorm Trace Elements Serum Level-2	(1.67 ± 0.10) × 10^–7^	(1.59 ± 0.11) × 10^–7^	4.79

### Comparing the Developed Sensor to Alternative Spectroscopic and Fluorometric Methods

Nickel is classified as a trace element because it is present in very small quantities in the environment. Because of these low concentrations, advanced instrumental analysis methods are essential for detection. Various techniques are employed to assess the nickel content in different samples. Based on the literature review, Kanchi et al. developed an optimized method to detect Ni(II) in grape samples using complexation with APDC and AMDC, followed by differential pulse polarography [25]. In the same year, Muthuselvi developed a spectrophotometric method using nicotinohydroxamic acid (NHA) and Triton X-100 at pH 9.0 to measure Ni(II) in tap water and alloys, showing high sensitivity and adherence to Beer’s law [26]. Furthermore, Khan et al. introduce a novel fluorescent probe (Pyr-1) characterized by spectroscopic techniques and supported by DFT studies, which displayed low cytotoxicity and strong fluorescence in living cells, showcasing its potential as a chemosensor for Ni(II) in biological environments [27]. In a recent study, Gu et al. presented a sensitive and selective method that utilizes orange photoluminescent carbon dots (O-CDs) for trace-level Ni(II) determination in lettuce and chocolate samples [28]. Within the scope of this study, the developed method has been rigorously compared with existing methods in the literature based on seven distinct parameters, with detailed results provided in Table 3.

**Table 3:** A comparative analysis of the developed method with selected techniques for Ni(II) determination

Method	Detection Limit (mol L^−1^)	Linear Range (mol L^−1^)	Time (sec)	System	Recovery%	Reference
Differential Pulse Polarographic Catalytic Hydrogen Wave Technique	1.8 × 10^–6^	3.2 × 10^–6^ – 1.17 × 10^–3^	7.5 min	Red grape	98.9–100	[25]
Spectrophotometric	9.17 × 10^–6^	2.69 × 10^–5^- 1.46 × 10^–4^	-	Tap water	99.4–101.2	[26]
Spectrofluorimetric	2.5 × 10^–7^	0–4 × 10^–6^	1 h	Human cervica cancer cell	-	[27]
Spectrofluorimetric	4.22 × 10^–8^	5.00 × 10^–6^- 5.00 × 10^–5^	-	Lettuce, chocolate	97.26- 100.90; 98.24–101.99	[28]
ICP-OES	1.07 × 10^–7^	1.24 × 10^–4^ – 5.66 × 10^–4^	90 s	Crude oil	-	[29]
Solvent-Assisted Dispersive Solid Phase Extraction-Flame Atomic Absorption Spectrophotometric	1.19 × 10^–8^	3.41 × 10^–8^ – 2.56 × 10^–6^	60 s	Tap water, well water, river water, and sea water	97–101	[30]
Spectrofluorimetric	4.81 × 10^–9^	1.70 × 10^–8^ – 3.41 × 10^–7^	20 s	Wastewater, blood serum, tap water	103.1–104.6	This study

## Conclusion

In conclusion, this study successfully developed and characterized a polymeric fluorescence sensor to detect Ni(II) ions. The sensor demonstrated high sensitivity and selectivity, with a detection limit of 4.81 × 10⁻⁹ mol L^−1^ and an optimal pH of 5.0. Structural and morphological analyses confirmed the prepared functional membranes, while fluorescence measurements established the sensor's ability to detect Ni(II) ions efficiently under specific conditions. The sensor's regeneration and reusability were validated, withstanding at least 150 cycles without significant performance loss and exhibiting long-term storage stability. Its selectivity against common interfering ions further highlights its practicality for Ni(II) detection in complex aqueous environments. Real-sample analyses, including wastewater, blood serum, and spiked tap water, confirmed the method’s accuracy, with recovery rates ranging from 103.1% to 104.6% and relative error values between 2.29% and 4.79% (95% confidence levels, *n* = 6).

Compared to existing methods, this approach offers significant advantages, such as cost-effectiveness, eco-friendliness, a rapid analysis time of 20 s, and the elimination of harmful solvents and extraction processes. These findings indicate that the developed sensor shows great promise as an alternative method for Ni(II) ion detection in various environmental and biological real samples, paving the way for further advancements in fluorescence-based sensing technologies.

## Supplementary Information

Below is the link to the electronic supplementary material.

Supplementary file1 (DOCX 144 KB)

## Data Availability

No datasets were generated or analysed during the current study.
