# Supplementary material for: Rapid and Precise Detection of Nickel(II) Ions Using a Fluorescent Polymeric Sensor
Source: J Fluoresc. 2025 Jul 1;35(11):11931–40. doi: 10.1007/s10895-025-04439-z (PMC12718274; doi:10.1007/s10895-025-04439-z)
Supplement: Supplementary file 1 — Supplementary file1 (DOCX 144 KB) [file 10895_2025_4439_MOESM1_ESM.docx]

**Supporting Information**

**Rapid and Precise Detection of Nickel(II) Ions Using a Fluorescent Polymeric Sensor**

Soner Çubuk^a,^ *, Tutku Demirtaş^a^, Belma Gjergjizi Nallbani ^a,b^, Memet Vezir Kahraman^a^ ^a^Department of Chemistry, Faculty of Sciences, Marmara University, 34722 Istanbul, Türkiye

^b^ UBT – Higher Education Institution, Faculty of Pharmacy, Lagjia Kalabria 10000 Prishtina, Republic of Kosovo

* Corresponding Authors

e-mail: sonercubuk@marmara.edu.tr

Tel: +90 216 777 3372

**Preparation of polymer-based fluorescence sensor**

Challenges such as cracking and disintegration in aqueous environments may arise following the preparation of polymeric membranes. To address these issues, the concentration and proportion of cross-linking monomers and the UV curing duration can be optimized to achieve the desired structural and functional properties. The formulation determined to be most effective in preventing such defects consists of 60% polyethylene glycol diacrylate (PEGDA), 40% trimethylolpropane triacrylate (TMPTA), 1% 2,5-dimercapto-1,3,4-thiadiazole (2SH), and 20% pentaerythritol tetrakis(3-mercapto propionate) (4SH), with 1% Camphorquinone (CQ) and 2% Darocure 1173 serving as photoinitiators.

The polymer mixture was placed into a Teflon mold with dimensions of 1.2 cm × 4.0 cm × 0.2 cm and subjected to UV irradiation (OSRAM 300 W, λmax = 365 nm) for 3 minutes to complete curing. Subsequently, the membranes were immersed in ultrapure water overnight to remove unreacted components, during which no swelling, disintegration, or morphological deformation was observed. The same study was also done with solutions at different pH values ​​and similar results were obtained. To enhance sensor performance, the membranes were dried for 24 hours in a lyophilizer, facilitating complete dehydration and the development of a porous structure suitable for further characterization and application.

**Identifying the Optimal pH Level for the Developed Sensor**

**
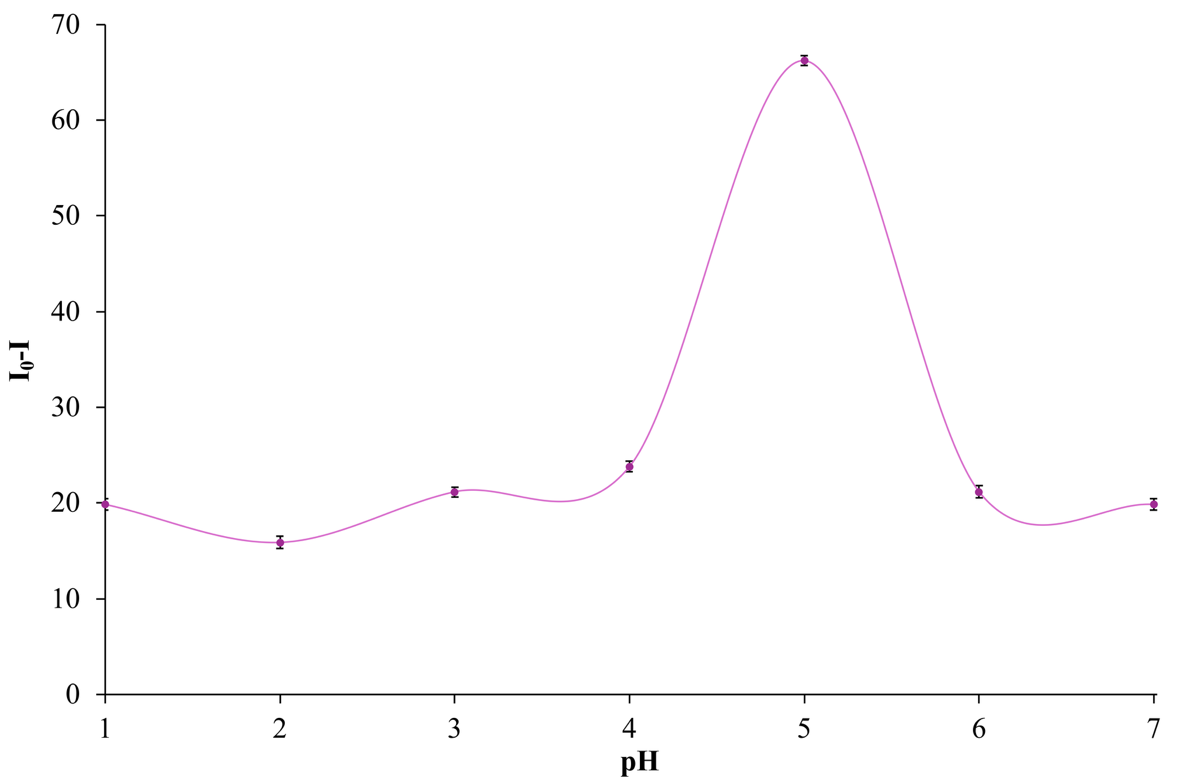
**

**Figure S1.** Influence of pH on fluorescence intensity.

**Determining the Response Time for the Developed Sensor**

**
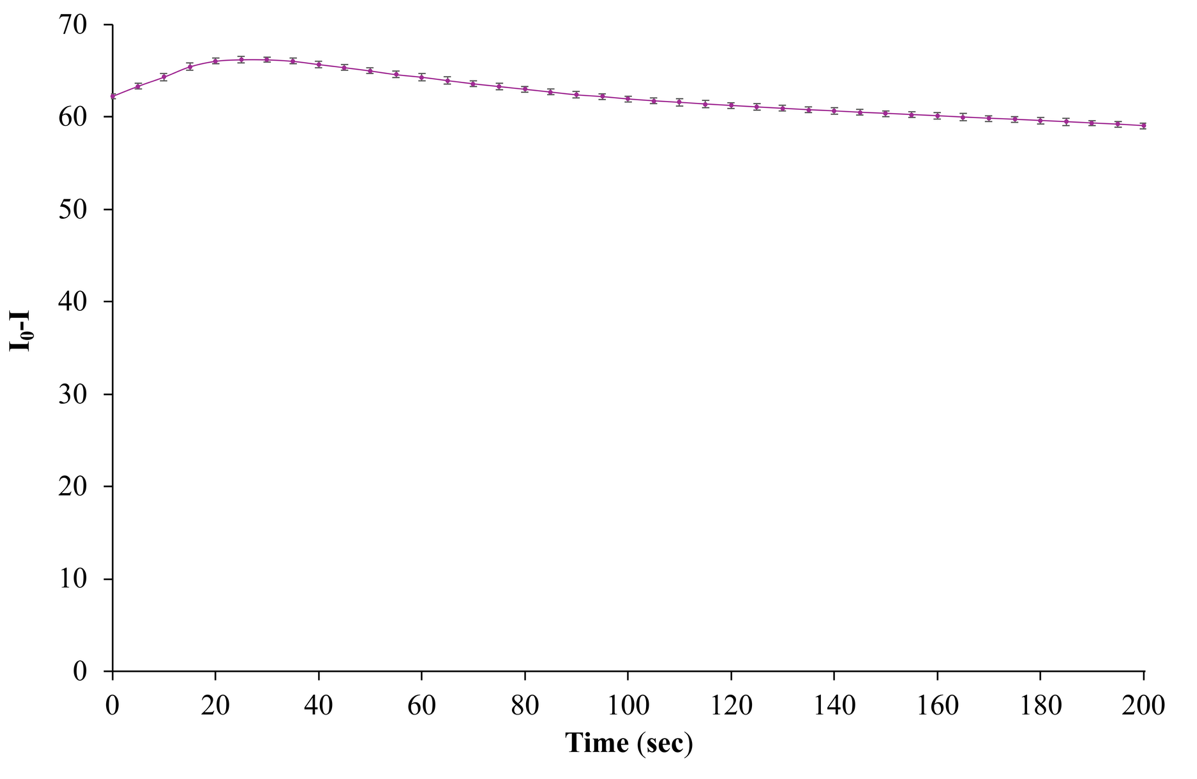
**

Figure S2. Temporal variation in fluorescence intensity of the sensor (pH: 5.0; C_Ni(II):_ 1.19 × 10⁻⁷ mol L^-1^).

**Table S1.**  The Results of Recovery Studies (95% confidence levels, n = 6).

| **Sample** | **Added Ni(II) concentration (mol L^-1^)** | **Current study**  **(mol L^-1^)** | **Recovery**  **(%)** |
| --- | --- | --- | --- |
| Tap water | - | (4.82 ±0.18) x10^-8^ | - |
|  | 4.26x10^-8^ | (9.24±0.12) x10^-8^ | 101.7 |
|  | 8.52x10^-8^ | (1.36±0.05) x10^-7^ | 101.9 |
|  | 1.70x10^-7^ | (6.69±0.08) x10^-7^ | 102.5 |
